# Molecular mechanisms and neural mediators of leptin action

**DOI:** 10.1101/gad.352550.124

**Published:** 2025-07-01

**Authors:** Cagri Bodur, Allison Duensing, Martin G. Myers

**Affiliations:** 1 Department of Internal Medicine, University of Michigan, Ann Arbor, Michigan 48019, USA;; 2 Department of Molecular and Integrative Physiology, University of Michigan, Ann Arbor, Michigan 48019, USA

**Keywords:** leptin receptor, JAK2, STAT3, obesity, hypothalamus

## Abstract

The adipose-derived hormone leptin signals the adequacy of body triglyceride stores to specialized leptin receptor (LepRb)-containing cells, which modulate physiology and behavior appropriately for the status of energy reserves. Decreased leptin action initiates a program that restrains a host of energy-intensive processes, promotes food seeking and consumption, and supports the continued availability of glucose and other metabolic fuels in the face of diminished fat stores. In addition to activating the STAT3-dependent transcriptional regulation that mediates most leptin action in vivo, LepRb mediates some leptin effects via a poorly understood second intracellular signaling pathway. Leptin also activates feedback pathways that restrain LepRb signaling in the face of high leptin, as in obesity. Leptin mediates most of its metabolic effects via multiple populations of *Lepr*-expressing hypothalamic neurons, each of which controls different aspects of leptin action. Although most of these neuron populations contribute only modestly to the control of food intake and body weight by leptin, *Glp1r*-expressing *Lepr* neurons inhibit *Agrp* neurons and strongly suppress feeding and body weight. Going forward, it will be important to define the potentially distinct intracellular responses to leptin for individual *Lepr* neuron populations, along with the cell type-specific roles for these responses in the physiologic effects of leptin.

## Abstract (precis)

In this review, Bodur et al. discuss the intricacies of leptin signaling and the neuronal populations that mediate the effects of leptin and its receptor. An understanding of how these pathways modulate food intake and body weight illuminates the significance of leptin signaling in controlling metabolism, physiology, and disease.

## Leptin and its receptor

Leptin is a 16 kDa monomeric peptide hormone that is secreted by adipocytes in approximate proportion to their triglyceride stores (Zhang et al. 1994; Pan and Myers 2018). In addition, insulin promotes leptin (*Lep*) gene expression and leptin synthesis (MacDougald et al. 1995; Bradley and Cheatham 1999). Because the secretion of leptin is constitutive, these effects increase circulating leptin. Conversely, sympathetic nervous system (SNS) activity generally inhibits leptin production (Couillard et al. 2002). Hence, leptin increases in states of nutritional repletion, whereas fasting and increased energy utilization can suppress leptin production more rapidly than fat stores decrease (Pérusse et al. 1997). Circulating leptin concentrations thus signal a combination of energy requirements and the status of body energy stores.

Leptin communicates this information about energy stores to systems that modulate physiology and behavior appropriately for the circumstances. Leptin not only suppresses hunger, it also promotes energy expenditure by augmenting basal metabolic rate, locomotor activity, and energy-intensive neuroendocrine processes, including via the reproductive and growth axes (Ahima et al. 1996; Haynes et al. 1997; Rosenbaum et al. 2005). Low leptin also augments some SNS responses during emergencies, thereby ensuring sufficient nutrient mobilization to support survival responses in the face of depleted energy stores (Flak et al. 2014, 2017). Leptin also diminishes anxiety, presumably to balance the potential for starvation against the risk of predation (Lu et al. 2006; Liu et al. 2010).

Consistently, leptin-deficient *Lep*^*ob/ob*^ (*ob/ob*) mice and rare cases of humans with inactivating *Lep* mutations display hyperphagia, decreased energy expenditure, obesity, impaired glycemic control, and reduced growth and reproductive function, among other phenotypes (Montague et al. 1997). Humans and rodents with lipodystrophy have diminished and dysfunctional adipose tissue and consequently produce little leptin (Shimomura et al. 1999; Oral et al. 2002). This leptin deficiency causes alterations similar to those observed in genetic leptin deficiency, though lipodystrophy results in ectopic lipid deposition rather than obesity. Exogenous leptin ameliorates many effects of lipodystrophy.

The ability of exogenous leptin to correct the obesity and other defects associated with leptin deficiency initially suggested the potential utility of leptin as a therapy for obesity (Maffei et al. 1995; Halaas et al. 1997). Individuals with obesity have high circulating leptin due to their increased fat mass, however, and exogenous leptin minimally alters food intake and body weight in most cases of obesity (Heymsfield et al. 1999). Hence, leptin plays crucial roles in signaling the inadequacy or adequacy of fat stores over the low to normal range (respectively) of leptin concentrations, while further increases in leptin with energy surfeit produce little additional weight-lowering effect.

Leptin acts by binding and activating its cell surface receptor (LepR, encoded by *Lepr* in rodents; here we use murine nomenclature and numbering throughout) (Tartaglia et al. 1995; Baumann et al. 1996; Chen et al. 1996; Chua et al. 1996). Alternative splicing of the *Lepr* transcript produces several mRNAs, each of which encodes unique LepR isoforms: a signaling-competent receptor with a long intracellular tail (LepRb; also known as ObRb), a variety of LepR isoforms with short intracellular tails (e.g., LepRa, LepRc, and LepRd), and a secreted isoform consisting of the LepR extracellular domain (LepRe) (Chung et al. 1996; Chua et al. 1997). Together, a soluble LepR extracellular domain produced by the proteolytic cleavage of membrane-bound LepR and LepRe represent the major circulating leptin binding proteins (Maamra et al. 2001; Ge et al. 2002). Although rapid increases in soluble LepR can sequester leptin and diminish leptin action acutely, soluble LepR appears to stabilize leptin over the long term (Zhang and Scarpace 2009; Lou et al. 2010).

**Figure 1.**
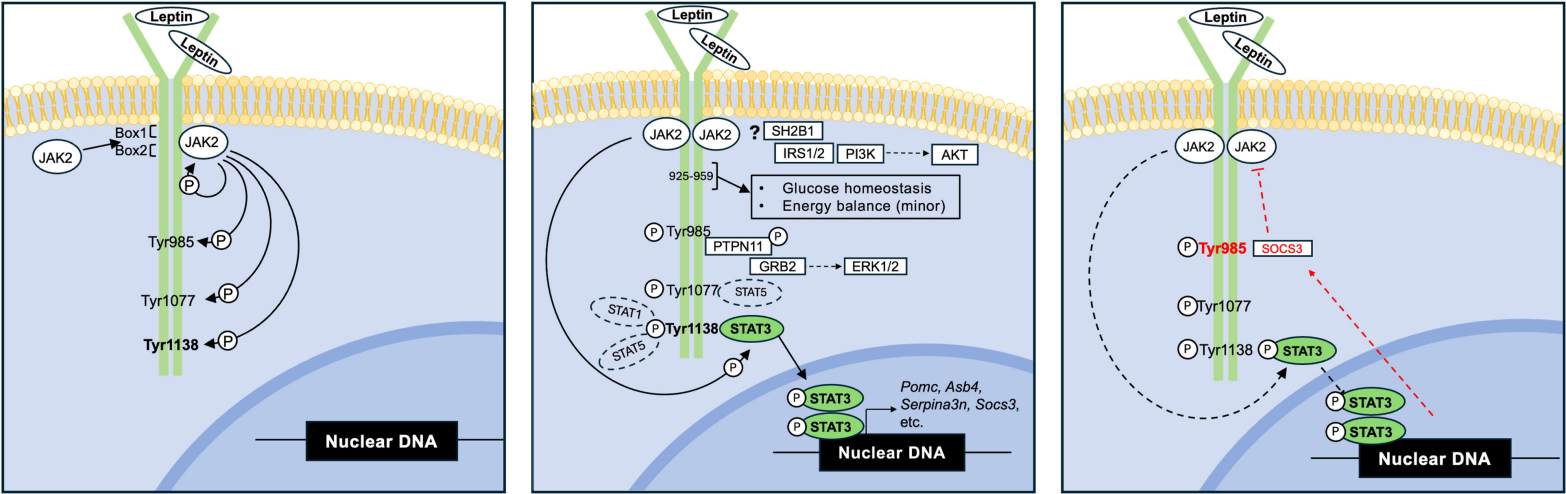
LepRb signaling. (*Left*) Leptin binding to LepRb activates the JAK2 tyrosine kinase, which constitutively associates with the membrane-proximal region of the LepRb intracellular tail via Box1 (866–877) and Box2 (909–921) motifs. Activated JAK2 phosphorylates three intracellular LepRb tyrosine residues: Tyr_985_, Tyr_1077_, and Tyr_1138_. (*Middle*) Each of these phosphorylated tyrosine residues (pTyr) recruits distinct SH2-containing effector proteins. pTyr_1077_ and pTyr_1138_ recruit STAT proteins, leading to their tyrosine phosphorylation, dimerization, and nuclear translocation/transcriptional activation. While pTyr_1077_ binds STAT5, pTyr_1138_ primarily recruits STAT3 (though it can also weakly recruit STAT1 and STAT5). While Tyr_1077_, STAT1, and STAT5 are dispensable for leptin action, Tyr_1138_ and STAT3 are required for most leptin action. pTyr_985_ recruits PTPN11 and SOCS3. Tyrosine phosphorylation of PTPN11 recruits GRB2, ultimately leading to the activation of ERK1/2 signaling. The sequences lying between LepRb residues 925 and 960 can mediate a second physiologically relevant signal that modulates blood glucose and mediates minor effects on energy balance. Candidates for this second LepRb signaling include the SH2B1 → IRS1/2 → PI3K/Akt pathway. (*Right*) The recruitment of SOCS3 to pTyr_985_ inhibits LepRb/JAK2 signaling as part of a feedback inhibition pathway.

Biochemical, cryo-EM, and molecular docking studies suggest that LepR dimerization on the cell membrane involves a two-step process (Fig. 1; Mancour et al. 2012; Saxton et al. 2023). Leptin initially binds a high-affinity site in the LepR extracellular cytokine homology region 2 to form a 1:1 complex. Subsequent interactions between leptin and the LepR IgG domain form a second low-affinity binding interface, leading to formation of a partially open 2:2 complex whereby one leptin peptide simultaneously binds two LepR molecules and a second leptin peptide contacts only one LepR.

## JAK2–STAT3 signaling by LepRb mediates leptin action

Like other type I cytokine receptors, LepR does not possess intrinsic enzymatic activity; rather, leptin-mediated LepR dimerization and conformational changes activate JAK2, a member of the Janus kinase family of cytoplasmic tyrosine kinases (Allison and Myers 2014). JAK2 noncovalently associates with LepRb via so-called “Box1” and “Box2” motifs in the membrane-proximal region of the intracellular domain (LepRb residues 866–877 and 909–921, respectively) (Fig. 1). The short LepR isoforms contain only Box1 but weakly activate JAK2 in overexpression systems (Uotani et al. 1999; Kloek et al. 2002). In contrast, LepRb contains both Box1 and Box2 and strongly activates JAK2, even at low endogenous levels of receptor and JAK2. Similarly, only LepRb contains the other intracellular motifs required for signal transduction. Hence, *db/db* mice, which lack only the LepRb isoform, exhibit a phenotype essentially identical to that of *ob/ob* animals (Chen et al. 1996; Chua et al. 1996).

Consistently, LepRb is restricted to several relatively small sets of neurons in the brain and a few cell types in the periphery (collectively, *Lepr* cells) that need to sense and respond to body energy stores and needs (Ghilardi et al. 1996; Mercer et al. 1996; Elmquist et al. 1998; Patterson et al. 2011). The exact physiologic role for the more widely expressed short LepR isoforms remains unclear, though proteolytic cleavage of these isoforms (especially from the liver) generates most circulating soluble LepR (Chung et al. 1996; Chua et al. 1997; Cohen et al. 2007).

Activated JAK2 phosphorylates the three tyrosine residues (Tyr_985_, Tyr_1077_, and Tyr_1138_) on the 302 amino acid intracellular tail of LepRb (Fig. 1; Allison and Myers 2014). These phosphorylated tyrosine (pTyr) residues serve as docking sites to recruit downstream signaling proteins that contain pTyr-binding elements like SH2 domains. Phosphorylated Tyr_1077_ and Tyr_1138_ (pTyr_1077_ and pTyr_1138_, respectively) recruit members of the SH2 domain-containing family of signal transducers and activators of transcription (STAT) proteins. The recruitment of STAT proteins by cytokine receptors results in their tyrosine phosphorylation (pSTAT), dimerization, and translocation to the nucleus to modulate target gene expression (Levy and Darnell 2002).

While pTyr_1077_ binds STAT5, pTyr_1138_ mainly recruits STAT3, though it can also weakly recruit STAT1 and STAT5, at least in cultured cells (Flak and Myers 2016). Ablation of *Stat3* in the CNS (or in *Lepr* cells) or the mutation of LepRb Tyr_1138_ in mice promotes hyperphagic obesity and other defects in leptin action, though these effects do not fully recapitulate the phenotype of LepRb deficiency (Bates et al. 2003; Gao et al. 2004; Jiang et al. 2008; Piper et al. 2008). Conversely, *Lepr* cell-specific expression of a constitutively active STAT3 modestly decreases adiposity in normal mice, as well as decreases body weight and improves glycemic control in *ob/ob* mice (Pan et al. 2019).

In contrast, ablation of *Stat5* from *Lepr* cells does not detectably alter leptin action; similarly, mutation of LepRb Tyr_1077_ minimally alters energy balance and glucose homeostasis (Patterson et al. 2012; Singireddy et al. 2013; Pan et al. 2019). Furthermore, ablating *Stat1* in *Lepr* cells does not alter leptin action on its own or exacerbate the phenotype of *Lepr*-specific *Stat3*-null mice (Pan et al. 2019). Hence, the recruitment of STAT3 to LepRb pTyr_1138_ represents the main STAT-dependent LepRb signaling pathway and mediates most leptin effects in vivo.

Deep sequencing of mRNA from hypothalamic *Lepr* neurons has defined the aggregate cell-autonomous transcriptional response to leptin, including the STAT3-dependent gene expression program (Allison et al. 2015, 2018). In addition to modulating the expression of several neuropeptides, leptin → STAT3 signaling prominently promotes the expression of several transcription factors (including immediate early genes), intracellular signaling molecules, and extracellular protease inhibitors. The roles played by the individual genes that comprise the overall transcriptional response to leptin remain undefined, however.

## Inhibitors of LepRb signaling

Phosphorylated LepRb Tyr_985_ (pTyr_985_) does not bind STAT proteins but rather recruits two different SH2 domain-containing proteins: the protein tyrosine phosphatase (PTP) PTPN11 (also known as SHPTP2 or SHP2) and the suppressor of cytokine signaling 3 (SOCS3) (Fig. 1; Banks et al. 2000; Bjørbæk et al. 2000). However, despite its PTP activity, PTPN11 does not alter the phosphorylation of JAK2 or STAT3 (Banks et al. 2000; Bjørbæk et al. 2001). Rather, pTyr_985_-dependent recruitment of PTPN11 promotes its tyrosine phosphorylation. Phosphorylated PTPN11 binds the SH2 domain-containing growth factor receptor bound protein 2 (GRB2), which activates the RAS → ERK1/2 pathway. In contrast to PTPN11, SOCS3 inhibits JAK2 (Marine et al. 1999; Sasaki et al. 1999), blunting the tyrosine phosphorylation of LepRb and its downstream signaling mediators (Bjørbæk et al. 1998, 1999; Pedroso et al. 2014). Indeed, ablation of *Socs3* in the CNS augments LepRb signaling in vivo, promoting leanness (Mori et al. 2004).

**Figure 2.**
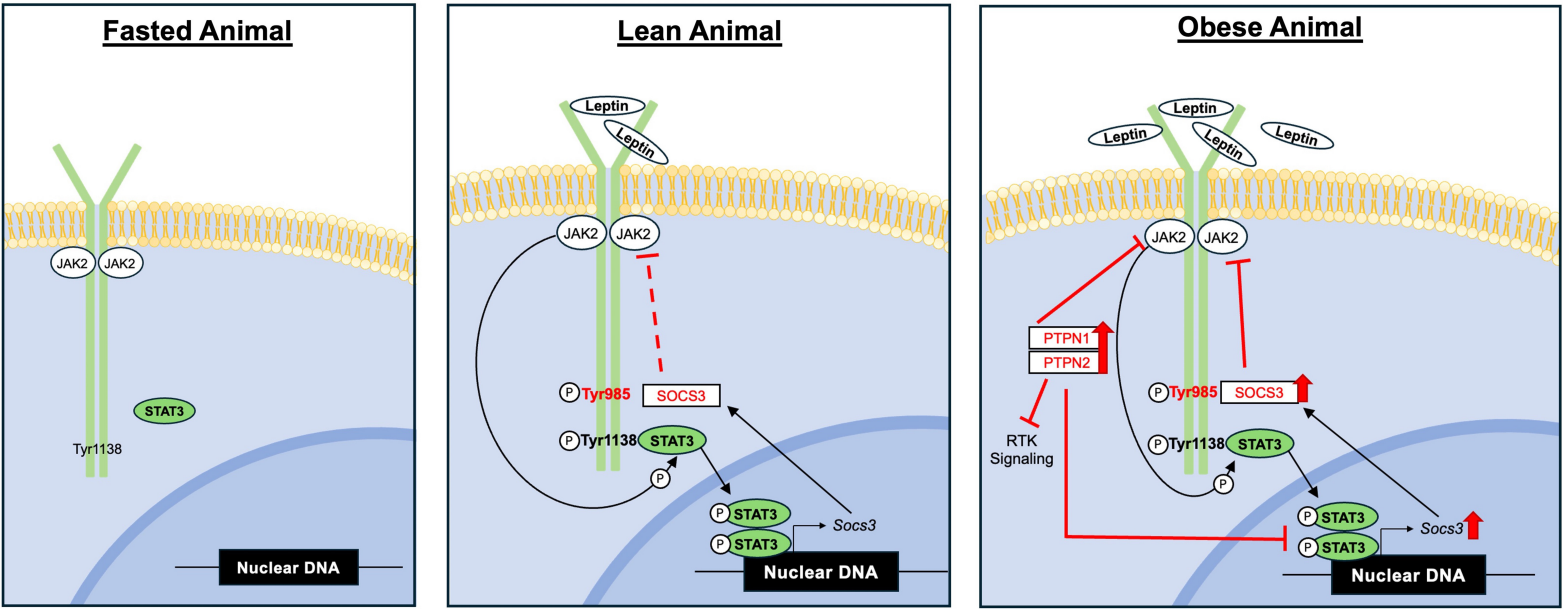
LepRb signaling across physiological states. (*Left*) Prolonged fasting or starvation depletes body fat stores, abrogating leptin production and thus reducing LepRb signaling. (*Middle*) Normal leptin concentrations in lean animals activate LepRb signaling, promoting the expression of STAT3-regulated genes, including *Socs3*. (*Right*) In obesity, elevated endogenous leptin increases LepRb signaling. Among other effects, this leads to the accumulation of inhibitors of LepRb signaling, including SOCS3, PTPN1, and TCPTP, thereby restraining the potential amplitude of LepRb signaling.

The finding that hypothalamic ERK1/2 activity is required for the leptin-mediated suppression of food intake and promotion of SNS outflow to brown adipose tissue (BAT) in vivo suggests the potential importance of the PTPN11 → ERK1/2 pathway in leptin action (Rahmouni et al. 2009). However, although deleting *Ptpn11* in subsets of hypothalamic *Lepr* neurons causes mild obesity and impaired glucose homeostasis, it does not attenuate the acute anorexic effects of leptin (Ke et al. 2007; Banno et al. 2010). Furthermore, mutation of LepRb Tyr_985_ in mice, which blocks the recruitment of PTPN11 and SOCS3 by LepRb, sensitizes leptin signaling and promotes leanness without otherwise altering leptin action (Björnholm et al. 2007). Hence, the dominant role for LepRb pTyr_985_ in vivo seems to be recruiting SOCS3 to inhibit LepRb/JAK2 signaling. Interestingly, LepRb → STAT3 signaling drives *Socs3* expression (Dunn et al. 2005). The LepRb pTyr_1138_ → STAT3-dependent accumulation of SOCS3 and the subsequent pTyr_985_-dependent inhibition of LepRb signaling by SOCS3 thus constitute a mechanism of feedback inhibition to restrain LepRb signaling when leptin concentrations are high (Figs. 1, 2).

The amplitude of LepRb signaling is restrained by several mechanisms, consistent with the threat posed by the undernutrition that might otherwise result from unopposed leptin action. In addition to SOCS3, the action of specific PTPs restrains leptin signaling by dephoshorylating pTyr residues within the LepRb signaling cascade (Fig. 2; Lund et al. 2005; Bence et al. 2006; Loh et al. 2011; Zhang et al. 2015). PTPN1 (also known as PTP1B) dephosphorylates JAK2 and STAT3 (Cheng et al. 2002; Kaszubska et al. 2002), and mice lacking *Ptpn1* throughout the body, in the brain, or specifically in *Lepr* cells display similar phenotypes of leptin hypersensitivity and leanness (Zabolotny et al. 2002; Bence et al. 2006; Tsou et al. 2012; Zhang et al. 2015). PTPN2 (also known as T-cell PTP [TCPTP]) promotes the dephosphorylation of pSTAT3 (Zhang et al. 2015). As for *Ptpn1*, neuron-specific deletion of *Ptpn2* leads to leptin hypersensitivity and leanness (Loh et al. 2011).

High endogenous leptin concentrations fail to reverse obesity, and treating obese patients or high-fat diet-induced obese (DIO) animals with exogenous leptin does little to alter food intake and body weight (Münzberg and Myers 2005; Myers et al. 2010; Pan and Myers 2018). Because exogenous leptin decreases feeding and body weight in lean animals, these observations led to the proposal that “leptin resistance” underlies the development and maintenance of obesity (Myers et al. 2010).

Although this term can mean many different things, one version postulates that leptin resistance results from the attenuation of cellular LepRb signaling. Proposed mechanisms for this attenuation include the actions of SOCS3 and PTPs, which are increased in obesity (Fig. 2; Myers et al. 2010). Indeed, ablating these LepRb signaling inhibitors in mice increases leptin responsiveness and promotes leanness. Exposure to a high-fat diet (HFD) can still increase food intake, body weight, and adiposity in these animals, however, and blunts the response to exogenous leptin. Thus, these mechanisms cannot represent the only cause of obesity. Other potential mediators of impaired leptin action in obesity include obesity-induced inflammation, cellular endoplasmic reticulum stress, and LepRb deacetylation (Zhang et al. 2008; Ozcan et al. 2009; Wisse and Schwartz 2009; Thaler et al. 2012; Guan et al. 2024). Indeed, drugs that block these processes can decrease body weight and adiposity in DIO rodents (Lee et al. 2016; Guan et al. 2024).

Although exogenous leptin treatment of DIO animals promotes little further increase in pSTAT3, untreated DIO mice with elevated endogenous leptin display increased hypothalamic pSTAT3, increased expression of many leptin-stimulated genes, and decreased expression of some leptin-inhibited genes (Fig. 2; Münzberg et al. 2004; Myers et al. 2008). Thus, the aggregate LepRb signaling response is not decreased in DIO animals at baseline but rather increases in response to the elevated leptin in these animals, even though additional increases in leptin do little to further increase LepRb signaling. Distinct *Lepr* cell types respond differently to DIO, however (Münzberg et al. 2004; Simonds et al. 2014).

The minimal effect of exogenous leptin in DIO suggests that increasing leptin above certain concentrations provokes little additional leptin action. This is consistent with the notion that altering behavior and physiology in response to low leptin/inadequate energy stores represents the major physiologic role for leptin, whereas elevated leptin in states of nutritional surfeit minimally alters physiology and behavior. Furthermore, elevated leptin may contribute to the pathophysiology of obesity. Leptin overexpression in rodent models transiently decreases body weight but causes increased body weight and adiposity over the long term (Qiu et al. 2001; Scarpace et al. 2002), whereas normalizing (decreasing) free leptin concentrations in DIO animals can decrease body weight and improve glycemic control (Zhao et al. 2019; Zhao et al. 2020). Furthermore, increased leptin action on *Lepr* neurons in the dorsomedial hypothalamic nucleus (DMH) may contribute to elevated SNS outflow and blood pressure in obesity (Enriori et al. 2011; Simonds et al. 2014).

The notion that reducing leptin in DIO animals improves metabolic function might seem at odds with the finding that the in chow-fed *ob/+* heterozygous mice display reduced leptin concentrations, resulting in increased adiposity and mild insulin resistance (Flatt and Bailey 1981; Chung et al. 1998). Leptin in chow-fed *ob/+* animals is decreased within the physiologic range of concentrations, however, whereas lowering leptin availability in DIO animals brings free leptin concentrations into the high normal range.

## STAT3-independent LepRb signaling

Mice deficient in LepRb Tyr_1138_ → STAT3 signaling do not fully recapitulate the food intake and body weight effects observed in *ob/ob* or *db/db* animals; these animals also display some preservation of glycemic control, lean mass, and neuroendocrine function (Bates and Myers 2004; Bates et al. 2004, 2005; Jiang et al. 2008; Piper et al. 2008). Hence, LepRb mediates at least one pTyr_1138_ → STAT3-independent signal that contributes to leptin action (Fig. 1). Interestingly, mice lacking all LepRb phosphorylation sites, like those lacking Tyr_1138_ only, exhibit a phenotype milder than that of *db/db* animals (Jiang et al. 2008). Furthermore, deleting intracellular LepRb sequences (including all intracellular LepRb Tyr residues) COOH-terminal to residue 960 produces a phenotype less severe than for *db/db* animals, but truncating LepRb at intracellular residue 921 or 925 produces the full LepRb deficiency phenotype despite intact JAK2 activation (Robertson et al. 2010; Barnes et al. 2020). Hence, an unidentified intracellular pTyr-independent LepRb motif residing between residues 925 and 960 can mediate a physiologically relevant signal.

Although the pTyr-independent LepRb signaling mechanism remains to be definitively identified, one candidate pathway involves SH2-containing protein B1 (SH2B1), the insulin receptor substrate proteins IRS1 and IRS2 (IRS1/2), phosphatidylinositol 3-kinase (PI3K), and/or the mechanistic target of rapamycin (mTOR) (Rui 2014). These signals indicate energy influx and/or cellular energy repletion.

Exogenous leptin promotes a small increase in hypothalamic PI3K activity, whereas inhibiting hypothalamic PI3K activity blunts the ability of leptin to decrease food intak, suggesting potential roles for this pathway in leptin action (Niswender et al. 2001). However, the LepRb intracellular domain contains no obvious PI3K binding motifs and poorly (if at all) activates PI3K signaling in cultured cells; the activation of PI3K signaling by the insulin → insulin receptor (IR) → IRS1/2 pathway is orders of magnitude stronger. Nonetheless, SH2B1 binds JAK2 and can form a complex with JAK2 and IRS1/2 in vitro and in overexpressing cell systems, where it promotes LepRb-dependent PI3K signaling (Duan et al. 2004; Rui 2014). These findings, together with the obesity of mice and humans with damaging mutations in *Sh2b1* and in mice lacking *Sh2b1* in the brain, suggest potential roles for a LepRb/SH2B1/IRS1/2 → PI3K pathway in leptin action (Doche et al. 2012; Jiang et al. 2020b).

In contrast, the ablation of PI3K catalytic activity in *Lepr* neurons produces a complex phenotype with increased thermogenesis and energy expenditure, leading to a lean phenotype despite increased food intake (Al-Qassab et al. 2009; Hill et al. 2009). Other non-LepRb receptor tyrosine kinase systems (including IR, TrkA, and TrkB, which also participate in energy balance) also form complexes with SH2B1 and mediate PI3K signaling, however (Maures et al. 2007). Hence, it is possible that altering SH2B1 or PI3K in *Lepr* neurons produces phenotypes that result from interfering with signaling by multiple receptor systems. Furthermore, despite the requirement for PI3K-activated hypothalamic mTOR activity to mediate leptin action, leptin modulates mTOR in opposite directions in different subsets of *Lepr* neurons (Cota et al. 2006; Villanueva et al. 2009).

Leptin also suppresses the activity of hypothalamic AMP-dependent protein kinase (AMPK), a serine/threonine kinase that monitors cellular energy levels. AMPK inhibits ATP-consuming processes and activates ATP-producing processes in response to signals of cellular energy deficit (Minokoshi et al. 2004; Kahn et al. 2005). Because leptin signals whole-body energy repletion while AMPK signals cellular energy depletion, it would make teleologic sense for leptin to block AMPK action, at least under some circumstances. Interfering with AMPK signaling in distinct, oppositely acting, sets of *Lepr* neurons produces opposite effects on energy balance, however, suggesting that the interaction between LepRb and AMPK signaling and its ultimate physiologic consequences may be cell type-specific (Claret et al. 2007).

Hence, although the well-described mechanisms underlying LepRb → STAT3 signaling remain consistent from cell type to cell type, the mechanisms by which leptin modulates PI3K, AMPK, and mTOR signaling remain unclear and vary from cell type to cell type, as do their consequences for leptin action. Definitively understanding roles for these pathways in LepRb action will require identifying the LepRb moiety that recruits them and/or modulating direct LepRb signaling to these pathways in vivo.

## Cellular targets of leptin action

Although most *Lepr* cells reside in the brain, some lie in peripheral tissues, including gut, bone marrow, and certain peripheral nerves (Comazzetto et al. 2021; Deng et al. 2022). Bone marrow and gut *Lepr* cells appear to serve as “niche” cells that control the development of rapidly proliferating cell types (e.g., specialized lymphoid cells and gut epithelium) from local stem cells. Decreased leptin action on these *Lepr* niche cells presumably restrains stem cell proliferation and alters the direction of cellular differentiation, decreasing the energetic demands imposed by the rapid expansion of immune cells and gut epithelium.

Like malnourishment, leptin deficiency impairs immune function and increases mortality from infectious disease (Lord et al. 1998; Farooqi et al. 2007). This presumably reflects an evolutionary trade-off that balances the need to conserve energy by minimizing the energetic cost of immune cell proliferation in the face of inadequate fat stores against the need to mount a sufficiently robust immune response to prevent infection-associated lethality. Consistent with its role in signaling the adequacy of energy stores, leptin largely reverses immune dysfunction during starvation (Lord et al. 1998; Matarese et al. 2005). Similarly, leptin (or *Lepr*) deficiency impairs immune responses and increases mortality from infectious diseases, whereas elevated leptin augments autoimmune attack (Lord et al. 1998; Matarese et al. 2002; Farooqi et al. 2007). While peripheral leptin action, including in bone marrow niche cells, contributes to the trophic effects on the immune system, CNS leptin action and brain LepRb signaling also mediate some leptin effects on immune function (Tschöp et al. 2010; Comazzetto et al. 2021). Hence, CNS and peripheral leptin action collaborate to support immune function. Central and peripheral leptin signaling may also cooperate to augment SNS tone (Rahmouni et al. 2002).

Leptin efficiently enters the brain (Maness et al. 2000; Banks et al. 2002; Di Spiezio et al. 2018; Butiaeva et al. 2021; Duquenne et al. 2021; Uner et al. 2023) to act on *Lepr* neurons, which mediate most of the effects of endogenous leptin on energy balance and metabolism (Cohen et al. 2001; Chua et al. 2004). Within the brain, several nuclei in the hypothalamus contain the most *Lepr* neurons, though some groups of *Lepr* neurons reside in the midbrain, the hindbrain, and a few other brain regions (Elmquist et al. 1998; Patterson et al. 2011). Consistently, the hypothalamus mediates most of the brain's contribution to leptin action (Ring and Zeltser 2010). Within the hypothalamus, the ARC, DMH, ventral premammillary (PMv), and ventromedial (VMH) nuclei, along with the lateral hypothalamic area (LHA), contain the largest and densest collections of *Lepr* neurons (Elmquist et al. 1998; Patterson et al. 2011). The preoptic area, zona incerta, and a few other hypothalamic areas also contain *Lepr* neurons.

## Leptin, the ARC, and the hypothalamic melanocortin system

**Figure 3.**
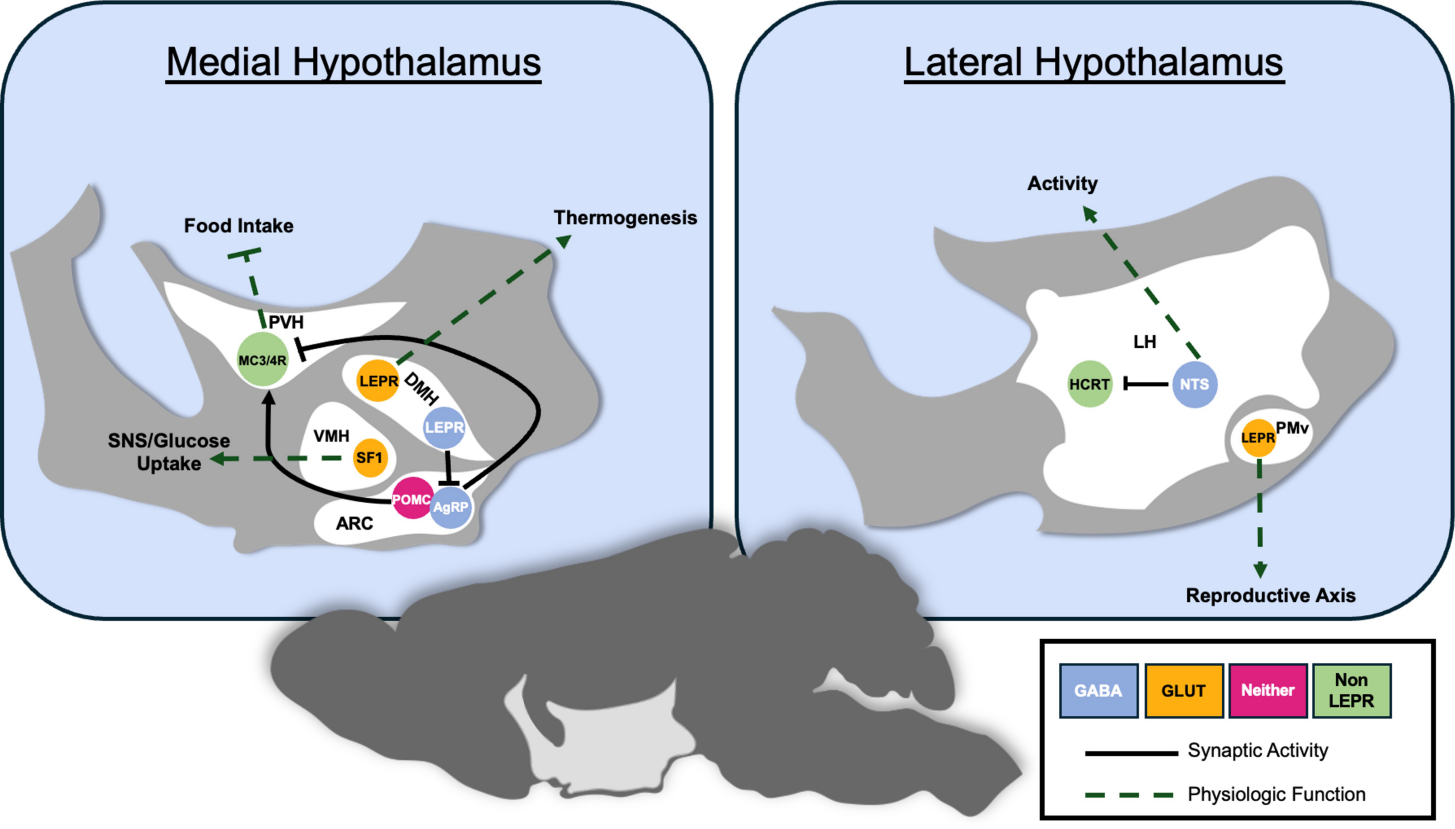
Hypothalamic mediators of leptin action. Shown is a cartoon depicting the best-understood hypothalamic *Lepr* neurons and their functions (other populations of Lepr neurons whose function is less well understood are not shown). In the medial hypothalamus, ARC POMC and AgRP neurons inhibit and stimulate feeding, respectively, in large part due to their actions on non-*Lepr* MC4R neurons in the PVN. Although POMC and AgRP neurons express *Lepr*, the inhibition of AgRP neurons by GLP1R-containing *Lepr* neurons represents a major mechanism by which leptin suppresses feeding. SF1-containing VMH *Lepr* neurons promote SNS-dependent glucose uptake and metabolism in skeletal muscle, and BAT and dDMH *Lepr* neurons (including those that express *Prlh*) promote thermogenesis and energy expenditure. More laterally, PMv *Lepr* neurons support neuroendocrine reproductive function, and LHA *Lepr* neurons, including those that contain NTS, inhibit LHA HCRT neurons and project to the VTA and other caudal regions to promote locomotor activity.

Serendipitously, the identification of leptin and LepRb closely followed the recognition of the hypothalamic melanocortin (MC) system, enabling the rapid recognition that leptin regulates the MC system (Fig. 3; Roselli-Rehfuss et al. 1993; Low et al. 1994; Ollmann et al. 1997; Atasoy et al. 2012). The hypothalamic MC circuitry consists of two sets of ARC neurons and their downstream targets. One of these ARC populations expresses pro-opiomelanocortin (*Pomc*), which produces a large precursor peptide that is cleaved into several smaller active neuropeptides, including forms of melanocyte-stimulating hormone (MSH). Although ablating ARC *Pomc* causes hyperphagic obesity, few *Pomc* neurons contain fast amino acid transmitters (e.g., GABA or glutamate), and artificially activating these cells provokes relatively modest effects on feeding (Lam et al. 2015; Campbell et al. 2017; Jiang et al. 2020a). Hence, the action of MSH peptides (rather than signaling by amino acid transmitters such as GABA and glutamate) presumably mediates most anorectic effects of *Pomc* neurons.

The brain contains two MC receptors: MC3R and MC4R (Allison and Myers 2014; Myers et al. 2021). Interfering with *Mc4r* or ARC *Pomc* neurons leads to dramatic hyperphagic obesity (Fan et al. 1997). In contrast, loss of *Mc3r* in mice produces a more complicated phenotype, with alterations in energy expenditure and feeding rheostasis that combine to produce a relatively mild late-onset obesity (Butler et al. 2000). Hence, ARC *Pomc* neurons and their *Mc4r*-expressing targets play crucial roles in the control of feeding and body weight; *Mc4r* neurons in the paraventricular hypothalamic nucleus (PVH) mediate major MC-dependent feeding effects (Shah et al. 2014).

A distinct set of ARC neurons expresses agouti-related peptide (*Agrp*), which inhibits signaling by MC3R and MC4R (Ollmann et al. 1997). ARC *Agrp* neurons also contain the inhibitory neurotransmitter GABA and the inhibitory neuropeptide Y (NPY) (Broberger et al. 1998). Hence, *Agrp* cells can suppress the overall activity of downstream neurons as well as block MC receptor signaling via AgRP. Interestingly, although activating *Agrp* neurons drives food seeking and profound hyperphagia, blocking their function minimally alters food intake or body weight under many conditions (Krashes et al. 2011; Wang et al. 2021; Cai et al. 2023). Developmental compensation may contribute to this lack of phenotype, however (Gropp et al. 2005; Luquet et al. 2005).

Both *Pomc* and *Agrp* neurons contain LepRb (Elias et al. 1999). Leptin increases the activity of *Pomc* neurons, and LepRb → STAT3 signaling augments *Pomc* expression (Mizuno et al. 1998; Elias et al. 1999). Conversely, leptin hyperpolarizes *Agrp* neurons and decreases their expression of *Agrp* and *Npy*, though this appears to be at least partly independent of STAT3 signaling (Cowley et al. 1999; Cone et al. 2001). Furthermore, interfering with the function of *Agrp* neurons attenuates the phenotype of *ob/ob* mice (Wu et al. 2012). Hence, many in the field initially expected that LepRb in *Pomc* and/or *Agrp* neurons would account for most of the control of feeding by leptin. Ablating *Lepr* from *Agrp* and/or *Pomc* neurons produces only mild obesity, however, though these manipulations impair glycemic control and other metabolic parameters (Balthasar et al. 2004; van de Wall et al. 2008; Berglund et al. 2012; Rupp et al. 2018).

## Hypothalamic leptin action outside of the ARC

Next to the ARC, the PMv contains the most *Lepr* neurons (Elmquist et al. 1998; Patterson et al. 2011). These glutamatergic *Lepr* cells project through the ventrolateral control column to terminate in the POA and surrounding regions (Leshan et al. 2009). In addition to their control by leptin, these neurons are activated by sex pheromones and promote neuroendocrine reproductive axis activity (Fig. 3; Hill et al. 2008; Donato et al. 2009). Ablating PMv *Lepr* impairs reproductive function but does not alter food intake, energy expenditure, or body weight (Hill et al. 2008; Donato et al. 2009, 2011).

The LHA *Lepr* neurons are mainly GABAergic, and many express various combinations of neurotensin (*Nts*), corticotropin-releasing hormone (*Crh*), and galanin (*Gal*); this population of neurons is centered in the perifornical area and extends into the lateral portion of the DMH (Leinninger et al. 2011; Laque et al. 2013; Goforth et al. 2014). Some LHA *Lepr* neurons also express growth hormone receptor (*Ghr*) or single-minded 1 (*Sim1*) (Cady et al. 2017; Cakir et al. 2019). The LHA contains neurons that project into the ventral tegmental area (VTA) and other regions to modulate the mesolimbic dopamine (MLDA) system and modify locomotor activity, reward, and consummatory behaviors (Fig. 3; Leinninger et al. 2011). *Nts*-expressing LHA *Lepr* neurons modulate the MLDA system (via direct VTA projections as well as indirectly by inhibiting LHA hypocretin [*Hcrt*] neurons) (Goforth et al. 2014). Ablating *Lepr* from *Nts* cells decreases locomotor activity and the response to certain drugs of abuse but minimally alters feeding (Leinninger et al. 2011). Similarly, ablating *Lepr* from *Ghr* or *Sim1* neurons reduces energy expenditure without impacting feeding (Cady et al. 2017; Cakir et al. 2019).

VMH *Lepr* neurons are glutamatergic and express steroidogenic factor 1 (*Sf1*; *Nr5a1*) (Fig. 3; Dhillon et al. 2006). They reside mainly in the dorsomedial portion of the VMH (dmVMH), which contains a heterogeneous mixture of neuron populations that control distinct SNS-dependent processes, including hepatic glucose production, glucose uptake into muscle and BAT, and energy expenditure (Affinati et al. 2021). Interfering with the function of VMH *Lepr* neurons or ablating *Lepr* from the VMH minimally alters food intake or glucose production but attenuates glucose uptake and energy expenditure, leading to increased adiposity in mice fed a high-fat diet (HFD) (Dhillon et al. 2006; Bingham et al. 2008; Sabatini et al. 2021; Sutton et al. 2021). POA *Lepr* neurons, while far less studied, also modulate energy expenditure (Yu et al. 2016, 2018).

The DMH contains multiple sets of *Lepr* neurons—some glutamatergic and some GABAergic—that are distributed across several regions of this large and diverse nucleus (Fig. 3; Rezai-Zadeh et al. 2014; Campbell et al. 2017). Activating GABAergic ARC-projecting and glutamatergic raphe pallidus-projecting *Lepr* neurons in the dorsal DMH (dDMH) increases thermogenesis and energy expenditure (Rezai-Zadeh et al. 2014; Francois et al. 2025). Consistently, ablating *Lepr* either from the relatively small population of prolactin-releasing hormone (*Prlh*)-expressing dDMH neurons or from *Brs3*-expressing neurons that span the DMH and LHA decreases energy expenditure, as does the stereotaxic ablation of DMH *Lepr* expression (Dodd et al. 2014; Rezai-Zadeh et al. 2014; Piñol et al. 2018; Faber et al. 2021). This latter maneuver can also provoke a short-lived increase in food intake, however, and these animals rapidly become obese. Thus, although many DMH *Lepr* neurons primarily control energy expenditure, one or more types of DMH *Lepr* neurons must contribute to the control of food intake. Indeed, ventral DMH (vDMH) targeted silencing of *Lepr* neurons increases food intake and promotes obesity, suggesting that some populations of *Lepr* neurons in this region modulate feeding (Faber et al. 2021).

## Neurotransmitter phenotypes of *Lepr* neurons that control feeding

Examining roles for direct leptin action on neurons that contain specific neurotransmitters revealed that although ablating *Lepr* from glutamatergic neurons decreases energy expenditure and promotes mild obesity, ablating *Lepr* from GABAergic cells produces hyperphagic obesity approaching that of *db/db* mice (Vong et al. 2011). Ablating *Lepr* from nitric oxide synthase-1 (*Nos1*)-expressing (NOSergic) neurons promotes a similar phenotype (Leshan et al. 2012).

GABAergic and NOSergic *Lepr* neurons represent large, widely distributed compilations of neurons, however (Vong et al. 2011; Leshan et al. 2012). Hence, these results could be interpreted to indicate that recapitulating the *db/db* phenotype requires interfering with direct leptin action across large numbers and multiple types of *Lepr* neurons. Importantly, however, ablation of *Lepr* from *Pdyn* or *Htr2c* neurons, each of which affects large swaths of *Lepr* neurons across many hypothalamic nuclei, produces no effect on feeding and little metabolic phenotype (Rupp et al. 2018). Thus, the total number of neurons from which *Lepr* is ablated matters less than the specific types of neurons targeted (Pan et al. 2018; Rupp et al. 2018).

*Agrp* neurons receive GABAergic innervation from vDMH LepRb neurons, and activating vDMH GABA or *Lepr* neurons inhibits *Agrp* neurons and blunts feeding (Garfield et al. 2016). Furthermore, food presentation activates vDMH *Lepr* neurons, thereby inhibiting *Agrp* neurons (Aitken et al. 2024). These vDMH neurons may also relay signals from the gut and hindbrain to inhibit *Agrp* neurons (Garfield et al. 2016; Aitken et al. 2024). Together with the finding that interfering with vDMH *Lepr* neurons increases feeding and body weight (Faber et al. 2021), these observations suggested the existence of a molecularly undefined population of GABAergic (and potentially NOSergic) vDMH *Lepr* neurons by which leptin indirectly inhibits *Agrp* neurons and suppresses feeding (Rupp et al. 2018).

## Molecularly defined populations of hypothalamic *Lepr* neurons

The foregoing analyses of *Lepr* neurons involved manipulating groups of neurons defined by their expression of neurotransmitters or other genes that researchers guessed might identify important sets of *Lepr* neurons. Many of these are unlikely to represent functionally unique populations of neurons, however. Fortunately, recent single-cell or single-nucleus RNA sequencing (scRNA-seq and snRNA-seq, respectively) analyses have enabled the unbiased identification of neuron populations that contain similar gene expression signatures and thus likely mediate similar functions (Campbell et al. 2017; Steuernagel et al. 2022; Rupp et al. 2023).

An early scRNA-seq analysis of the mbHypo not only demonstrated that *Pomc-*, *Agrp*-, and *Ghrh*-expressing *Lepr* neurons represent bioinformatically unique classes of ARC cells but also identified two novel groups of *Lepr*-expressing neurons (marked by the expression of *Trh* and *Tbx19*, respectively) (Campbell et al. 2017). This analysis also identified a somatostatin (*Sst*)-expressing neuron population in the tuberal nucleus (TU). Although they do not contain *Lepr*, TU *Sst* cells are activated by desirable food (e.g., chocolate) and drive food intake (Luo et al. 2018; Mohammad et al. 2021).

Purifying nuclei from hypothalamic *Lepr* neurons and subjecting them to snRNA-seq analysis also confirmed that several previously studied groups of *Lepr* neurons represent discrete neuron populations, including those marked by *Pomc*, *Agrp*, *Ghrh*, *Nr5a1* (VMH), *Irx5* (PMv), and *Nts/Crh/Gal* (LHA/lateral DMH) (Rupp et al. 2023). This analysis also identified the *Trh*- and *Tbx19*-expressing *Lepr* neuron populations detected previously in the mbHypo scRNA-seq (these populations are also marked by *Glp1r* and *Pirt*, respectively). These cell types are largely conserved across species. snRNA-seq of *Lepr* neurons identified no TU *Sst*-containing *Lepr* population, suggesting that if leptin modulates TU *Sst* neurons, it must do so indirectly.

Both *Trh/Glp1r*-expressing and *Tbx19/Pirt*-expressing *Lepr* neurons (referred to here as *Lepr/Glp1r* and *Lepr/Pirt* neurons, respectively) are GABAergic and express *Nos1* (Rupp et al. 2023). *Lepr/Pirt* neurons reside exclusively in the ARC, and their roles (along with roles for a few other newly defined populations of hypothalamic *Lepr* neurons) remain to be determined. The *Lepr/Glp1r* neuron population forms a continuum of cells extending from the caudal ARC into the vDMH (Fig. 3; Rupp et al. 2023) and thus meets the expected criteria for the putative population of *Lepr* neurons crucial for the leptin-mediated inhibition of *Agrp* neurons and suppression of food intake. Indeed, ablating *Lepr* from *Glp1r* neurons promotes substantial hyperphagic obesity. Interestingly, these neurons also represent a point of convergence for the effects of leptin and GLP1R agonism (Polex-Wolf et al. 2024). Furthermore, several recent studies that largely target *Lepr/Glp1r* cells demonstrate that these cells directly innervate and inhibit *Agrp* neurons, suppressing food intake (Kim et al. 2024; Polex-Wolf et al. 2024; Tan et al. 2024; Webster et al. 2024). Because the DMH (including DMH *Lepr* neurons specifically) and *Agrp* neurons are required for the circadian entrainment of food intake (Mieda et al. 2006; Tan et al. 2014; Faber et al. 2021), it is tempting to speculate that *Lepr/Glp1r* neurons also participate in this process.

## Functions for *Lepr* neurons outside of the hypothalamus

Most *Lepr* neurons that lie outside of the hypothalamus have been defined solely by their anatomic location, as most have not been molecularly defined in the manner of hypothalamic *Lepr* neurons. The dorsal vagal complex (DVC; including the area postrema [AP], nucleus of the solitary tract [NTS], and dorsal motor nucleus of the vagus [DMV]) in the caudal medulla represents the exception to this rule (Dowsett et al. 2021; Ludwig et al. 2021). The AP and NTS collect and process a host of interoceptive data from the body and relay this information rostrally to control behavior, as well as to the DMV to modulate parasympathetic outflow to appropriate target organs (Grill and Hayes 2012).

Within the DVC, the NTS and DMV contain *Lepr* cells (Huo et al. 2008; Do et al. 2020; Ludwig et al. 2021). The NTS contains two main glutamatergic *Lepr* neuron populations: One (marked by preproglucagon [*Gcg*] expression) controls feeding, and the other (marked by galanin [*Gal*] expression) modulates respiratory drive. Although *Lepr/Gcg* neurons receive information about nutrients in the gut and play important roles in the restraint of food intake, ablating *Lepr* from these cells or from the entire NTS minimally alters feeding or energy balance (Scott et al. 2011; Kreisler and Rinaman 2016; Brierley et al. 2021). Deleting NTS *Lepr* and/or modulating the NTS *Lepr/Gal* neurons alters the control of breathing, however (Do et al. 2020).

Rostral to the NTS in the pons, the parabrachial nucleus (PBN) and the periaqueductal gray (PAG) contain cholecystokinin (*Cck*)-expressing *Lepr* neurons (Flak et al. 2014, 2017). These neurons are inhibited by leptin and activated by noxious stimuli (PAG) and signals of hypoglycemia (PBN). Both PAG and PBN *Lepr* neurons act via the VMH to promote SNS outflow and potentiate hepatic glucose production in response to specific stimuli. Hence, decreased leptin action on PBN and PAG *Lepr* neurons ensures adequate glucose mobilization in the face of life-threatening emergencies when energy stores are low and more difficult to mobilize.

The midbrain also contains *Lepr* neurons, including in the dorsal raphe (DR), VTA, and substantia nigra (SN) (Elmquist et al. 1998; Patterson et al. 2011). Although the DR is best known for its serotonergic neurons, like most VTA and SN *Lepr* neurons, DR *Lepr* neurons are dopaminergic (Lam et al. 2011). Leptin action on these midbrain cells does little to modulate feeding but may alter stress- and anxiety-related behaviors. Other extrahypothalamic *Lepr* neuron populations remain to be molecularly defined and studied.

## Future directions

Although we have learned a great deal about the molecular and neural mediators of leptin action since the discovery of leptin and LepRb more than three decades ago, there remains much that we do not understand. While the bioinformatically defined *Lepr* neuron clusters studied to date appear to represent functionally coherent cell populations, single-cell transcriptomic analyses generate hypotheses about functional similarities among cells with similar transcriptional signatures, and these hypotheses must be tested. Hence, it will be important to define the physiologic roles played by a host of relatively unstudied *Lepr* neuron populations, including *Lepr/Pirt* cells.

Although the combined use of cre-expressing rodent models and stereotaxic injections has revealed a great deal about spatially defined sets of *Lepr* neurons, many regions contain multiple bioinformatically defined *Lepr* neuron populations that can only be manipulated in isolation by increasing the specificity of the genetic reagents with which we target them. Combining distinct transgenic alleles, each of which expresses different recombinases (e.g., Cre, Flp, and Dre), with genetic or viral tools that are sensitive to multiple recombinases can offer such specificity (Fenno et al. 2014; Biglari et al. 2021; Sabatini et al. 2021). Using these tools, we must define the roles for each *Lepr* neuron population, along with the downstream circuits by which they act. It will also be useful to apply additional genomic tools, including spatial techniques, to understand the anatomy and regulation of each *Lepr* neuron population.

Because STAT3-mediated transcriptional control underlies most leptin action, it will be important to understand the regulation of gene expression by leptin for the individual *Lepr* neuron populations, since different genes may dominate the leptin-mediated control of each set of neurons. Relatedly, we must determine how each population of *Lepr* neurons responds to DIO/hyperleptinemia and how these responses may contribute to the pathophysiology of obesity and obesity complications. Understanding the nature of the pTyr-independent LepRb signal and how it modulates *Lepr* neuron function will also be crucial.
